# Dietary inflammatory index and all-cause mortality in adults with COPD: a prospective cohort study from the NHANES 1999–2018

**DOI:** 10.3389/fnut.2024.1421450

**Published:** 2024-09-25

**Authors:** Tu-Lei Tian, Tian-Yu Zhi, Mei-Ling Xie, Ya-Lin Jiang, Xiang-Kun Qu

**Affiliations:** ^1^Department of Respiratory and Critical Care Medicine, The Affiliated Bozhou Hospital of Anhui Medical University, Bozhou, Anhui, China; ^2^Department of Clinical Medicine (5+3 Integrated Program), Shanxi Medical University, Taiyuan, China; ^3^Bengbu Medical University Graduate School, Bengbu, Anhui, China

**Keywords:** chronic obstructive pulmonary disease, dietary inflammatory index, mortality, cohort study, inflammation

## Abstract

**Background:**

Chronic inflammation is closely linked to Chronic Obstructive Pulmonary Disease (COPD); however, the impact of the Dietaryq Inflammatory Index (DII) on mortality among COPD patients remains uncertain.

**Objective:**

To assess the correlation between the DII and all-cause mortality in COPD patients using data from the National Health and Nutrition Examination Survey (NHANES).

**Methods:**

We conducted a retrospective cohort study on 1,820 COPD patients from the NHANES dataset (1999-2018). The influence of DII on mortality was evaluated using multivariate Cox regression, smoothing spline fitting, and threshold effect analysis. Additionally, Kaplan-Meier survival analysis was performed to compare survival curves among different DII groups. Subgroup analyses and E-values identified sensitive cohorts and assessed unmeasured confounding.

**Results:**

Over an average follow-up of 91 months, multivariate Cox regression models revealed a significant positive correlation between DII scores and mortality risk, with each unit increase in DII associated with a 10% higher risk of death (HR: 1.10, 95% CI: 1.03-1.16; P = 0.002). Among the DII tertiles, individuals in the second tertile (T2: 1.23-2.94) experienced a 67% increase in mortality risk compared to those in the lowest tertile (T1: -5.28-1.23) (HR: 1.67, 95% CI: 1.26-2.21; *p* < 0.001). The third tertile (T3) did not show a statistically significant increase in mortality risk (HR: 1.30, 95% CI: 0.98-1.72; *p*=0.074). A restricted cubic spline analysis indicated a significant nonlinear association between DII and all-cause mortality (*p* = 0.021). Threshold effect analysis further revealed that below a DII of 2.19, there was a significant increase in all-cause mortality risk (HR = 1.19, 95% CI: 1.07-1.33; *p* = 0.002), while at or above this threshold, the risk increase was not statistically significant (HR=0.89, 95% CI: 0.68-1.15; *p* = 0.380). Kaplan-Meier analysis revealed significant differences in survival curves among DII tertiles (*p* < 0.001), with the lowest DII tertile showing the highest survival probability. Both subgroup and sensitivity analyses confirmed the robustness of these findings.

**Conclusion:**

DII is positively correlated with mortality risk in COPD patients, showing nonlinear characteristics and threshold effects, underscoring its prognostic value.

## Introduction

Chronic Obstructive Pulmonary Disease (COPD) is a major chronic respiratory condition that has become a significant global public health challenge. According to the 2019 Global Burden of Disease report, approximately 212 million people worldwide are affected by COPD, accounting for 2.71% of the global population, and it ranks among the leading causes of death (1). COPD is characterized by high prevalence, severe mortality, and substantial economic burden, all of which significantly impact patients’ quality of life. In the United States alone, approximately 15.5 million adults have been diagnosed with COPD (2, 3), with the total economic burden estimated at $101 billion in 2020 (3). These figures underscore the widespread prevalence of COPD and its profound health and economic implications, highlighting the urgent need to optimize prevention and treatment strategies.

The pathogenesis of COPD is complex, with chronic inflammation serving as a pivotal mechanism. Long-term exposure to harmful stimuli such as tobacco smoke and air pollutants initiates abnormal inflammatory responses in the airways and alveoli, leading to airway remodeling and lung parenchyma destruction, which ultimately results in irreversible airflow limitation (4). During this process, inflammatory cells including macrophages and neutrophils are excessively activated, releasing substantial quantities of inflammatory mediators like interleukin-6 (IL-6) and tumor necrosis factor-alpha (TNF-α), exacerbating the inflammatory response and tissue damage (5). Additionally, oxidative stress and an imbalance between proteases and antiproteases further con-tribute to the development of COPD (4). Given the central role of chronic inflammation in COPD, controlling this inflammatory response has become a crucial aspect of current treatment and management strategies (6). Anti-inflammatory therapies, including the use of inhaled corticosteroids and phosphodiesterase-4 (PDE4) inhibitors, are commonly employed to reduce inflammation and slow disease progression (7). Previous studies have emphasized the impact of dietary factors on COPD. A review by Scoditti et al. (8) indicated a significant association between unhealthy dietary patterns, such as high fat and high sugar intake, and an increased risk of developing COPD. This perspective was further corroborated by a meta-analysis conducted by Zheng et al. (9), which not only confirmed the relationship between these dietary habits and the risk of COPD but also suggested potential implications for disease prognosis. Conversely, diets rich in fruits and vegetables have shown potential protective effects, possibly reducing the risk of developing COPD and improving the prognosis for those already affected (10, 11). These findings suggest that targeted dietary interventions aimed at reducing systemic inflammation could play a crucial role in the comprehensive management of COPD. This further underscore the importance of integrating dietary strategies with pharmacological treatments to more effectively manage chronic inflammation in COPD and improve long-term health outcomes for patients.

The Dietary Inflammatory Index (DII) is an innovative tool designed to assess the pro-inflammatory potential of a diet, calculated using 45 dietary components associated with inflammation. A higher score on the DII indicates a more potent pro-inflammatory effect of the diet (12). Extensive research has demonstrated that a diet with a high DII is closely linked to the risk and prognosis of chronic diseases such as cardiovascular diseases, diabetes, and cancer, as well as adverse outcomes in respiratory diseases including asthma and lung cancer (13–17). However, investigations into the relationship between the DII and COPD prognosis are still limited. A cross-sectional study indicated that COPD patients exhibit significantly higher DII scores compared to controls, suggesting a link between dietary inflammation and the onset of COPD (18). Nonetheless, there remains a shortage of long-term cohort studies assessing the impact of DII on COPD outcomes. Considering the central role of inflammation in COPD pathogenesis and the potential for dietary modulation, exploring the relationship between DII and COPD survival prognosis is crucial.

This study aims to utilize data from the National Health and Nutrition Examination Survey (NHANES) spanning 1999–2018, which includes 1,820 COPD patients, to explore the relationship between DII and all-cause mortality in COPD. We will analyze the dose–response relationship and subgroup differences, aiming to provide novel, evidence-based guidance for dietary management in COPD. We hypothesize that a diet high in DII is an independent risk factor for poor prognosis in COPD, and that the relationship between the two is nonlinear.

## Materials and methods

### Study design and participants

This study performed a detailed analysis of data from NHANES, overseen by the National Center for Health Statistics (NCHS) for the period 1999 to 2018. The primary aim was to extensively evaluate the health and nutritional status of the non-institutionalized civilian population in the United States. A complex stratified, multistage, a probabilistic sampling design was utilized to accurately mirror the health conditions prevalent across the nation. Participants provided detailed written informed consent prior to inclusion in the study (19). Additionally, all phases of the study’s design, data collection, and analysis strictly adhered to the Strengthening the Reporting of Observational Studies in Epidemiology (STROBE) guidelines, ensuring methodological rigor and compliance with ethical standards. The study protocols were approved by the Institutional Review Board of the NCHS (20).

**Figure 1:**
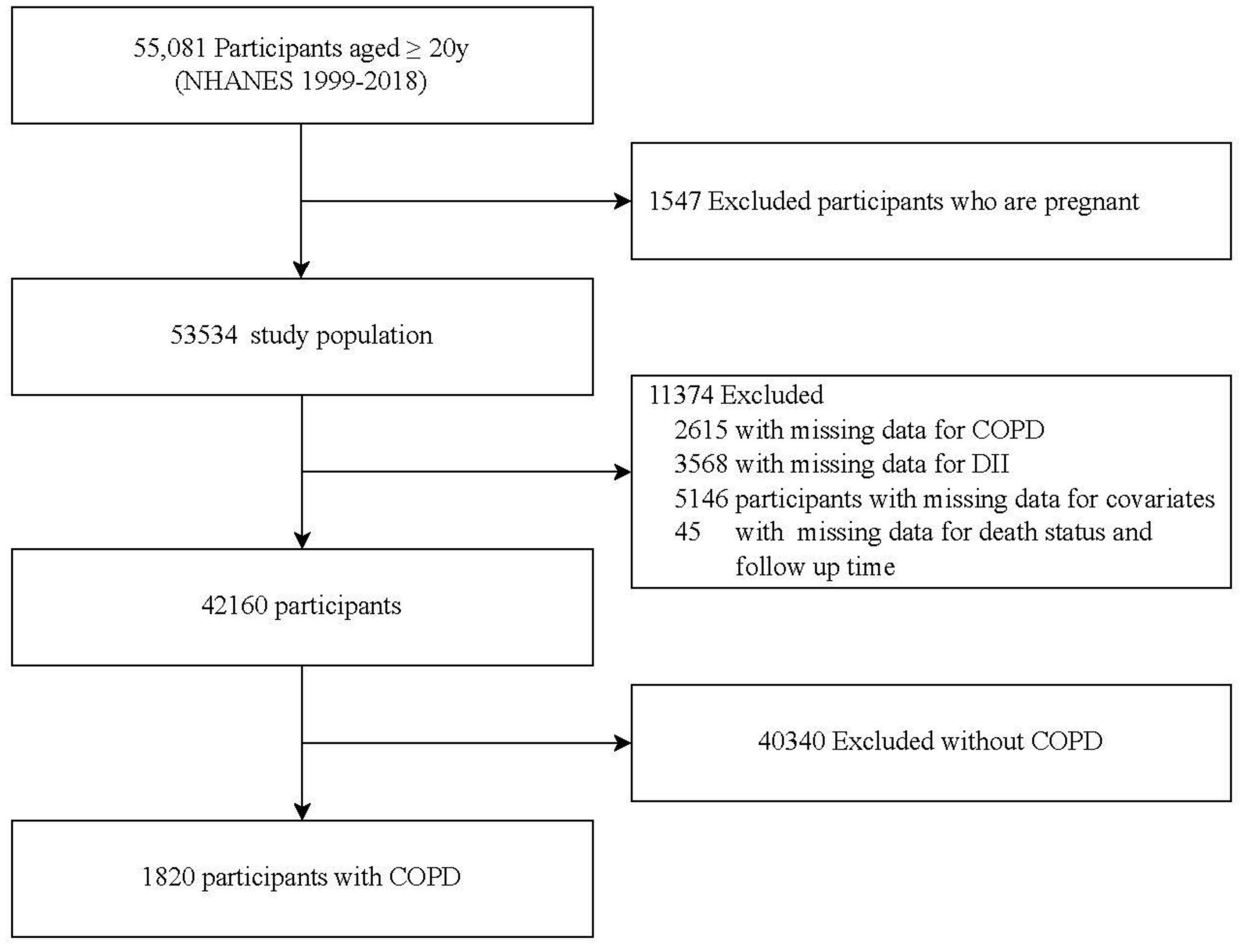
Flow chart for inclusion and exclusion of the study population.

This study initially screened 55,081 participants aged 20 years or older from the NHANES database for the period 1999 to 2018. Of these, 1,547 pregnant participants were first excluded. From the remaining 53,534 individuals, exclusions were as follows: 2,615 due to missing relevant COPD data; 3,568 for absence of DII data; 5,146 due to lacking other pertinent covariate data; and 45 for incomplete data on mortality status and follow-up time. Consequently, from the 42,160 eligible participants, 40,340 who did not have COPD were further excluded, leaving 1,820 patients with COPD for inclusion in the study. The study flowchart is presented in Figure 1.

### Definitions of COPD

The diagnosis of COPD was established based on one or more of the following criteria (21): (1) a post-bronchodilator FEV1/FVC ratio < 0.70; (2) a diagnosis of emphysema confirmed by a physician or other healthcare professional; or (3) for individuals aged 40 years or older, a history of smoking combined with chronic bronchitis and treatment with selective phosphodiesterase-4 (PDE-4) inhibitors, mast cell stabilizers, leukotriene modifiers, or inhaled corticosteroids.

### Measurement of DII

During the period from 1999 to 2018, dietary data from participants in the NHANES study were collected using two rounds of validated 24-h dietary recall surveys. The DII was utilized to quantify the inflammatory potential of dietary patterns, with the construction and validation of the DII as documented in prior research (12, 22). This study incorporated 28 dietary components in the DII calculation, which included carbohydrates, proteins, total fats, alcohol, fiber, cholesterol, saturated, monounsaturated, and polyunsaturated fatty acids, n-3 and n-6 fatty acids, niacin, vitamins A, B1 (thiamin), B2, B6, B12, C, D, and E, and minerals such as iron, magnesium, zinc, selenium, folate, carotenoids, caffeine, and energy. Despite the inclusion of only 28 parameters, the validity of these parameters has been confirmed by previous studies to ensure they comprehensively maintain the scientific accuracy and integrity of the DII framework (12, 23).

### Mortality ascertainment

Mortality data for NHANES participants aged 20 years and older were acquired through December 31, 2019, from the NHANES Public-Use Linked Mortality File, which is linked to the National Death Index (NDI) via a probabilistic algorithm.^1^ Maintained by the NCHS, this database provides reliable information on the causes of death in the U.S. population, with specific causes of death determined according to the International Classification of Diseases, Tenth Revision (ICD-10) (24, 25). Definitions of all-cause mortality encompass deaths from any cause. The median duration of follow-up for this research was 91 months. All participants were consistently monitored until death, loss of follow-up data, or the end of the study period on December 31, 2019.

### Covariates assessment

Based on existing research and clinical assessments (26, 27), confounding variables identified included age, sex, race, marital status, education level, the poverty income ratio (PIR), as well as body mass index (BMI), smoking habits, physical activity intensity, cardiovascular diseases (CVD), hypertension, and diabetes mellitus (DM). This study utilized self-reported data on race, classified into several categories: non-Hispanic white, non-Hispanic black, Mexican American, other Hispanic types, and additional racial groups. Marital status was classified into married, never married, living with partner, and other categories such as widowed, divorced, or separated. Education levels were organized from less than 9th grade to college graduate or higher. Economic status was quantified using the PIR, and BMI was calculated from clinical measurements of height and weight. Smoking status was segmented into never smokers (fewer than 100 lifetime cigarettes), ex-smokers (more than 100 cigarettes but has quit), and current smokers (more than 100 cigarettes and continuing) (28). Physical activity was assessed by the reported weekly duration of walking or cycling, chores, work, and recreational activities (29). The history of CVD was determined by self-reported incidents of CHD, angina, myocardial infarction, or stroke; Hypertension and diabetes mellitus diagnoses were also self-reported, with the latter including HbA1c levels exceeding 6.5%, a diagnosis by a physician, fasting plasma glucose ≥7.0 mmol/L, random or two-hour OGTT glucose ≥11.1 mmol/L, or the use of diabetes medications/insulin (30). More detailed information on these variables can be found on the NHANES website.^2^

### Statistical analyses

This study is a secondary analysis of publicly available datasets. We applied dietary weights for weighted analyses. For the combined analysis of NHANES 1999–2000 and 2001–2002 data, a four-year dietary weight set (WTDR4YR) was used, while for the 2003–2018 data, the dietary day-one sample weight set (WTDRD1) was applied. The sampling weights for 1999–2018 were calculated as follows: weights for 1999–2002 were 2/10 × WTDR4YR, and for all other years, 8/10 × WTDRD1. Categorical data were presented as unweighted numbers (weighted percentages), normally distributed continuous data were presented as weighted means (standard error [SE]), and skewed continuous variables were presented as weighted medians [IQR]. To assess differences between groups, we employed the following statistical methods: one-way analysis of variance (ANOVA) for normally distributed variables, the Kruskal-Wallis test for skewed variables, and the chi-square test for categorical variables. This comprehensive approach facilitated comparisons of continuous and categorical variables across different DII tertile groups.

A multifactorial Cox regression analysis was conducted to explore the relationship between DII and all-cause mortality, estimating its hazard ratio (HR) and 95% confidence interval (CI). Initially, DII was analyzed as a continuous variable and subsequently categorized into tertiles to examine its association with all-cause mortality among COPD patients. Non-adjusted Model was the unadjusted baseline model. Model 1 was adjusted for age, sex, race, PIR, marital status, and education level. Model 2 included additional adjustments for BMI, smoking status, physical activity time, CVD, hypertension, and diabetes, beyond the factors in Model 1 and treated categorical variables as continuous to test for linear trends.

A restricted cubic spline model, with the median value of the DII scores serving as the cutoff point, was used to examine the potential nonlinear dose–response relationship between DII and all-cause mortality in COPD patients. The model was adjusted for multivariate factors, and hazard ratio (HR) curves were generated at three knots. Subsequently, a segmented Cox regression model was developed to assess the relationship between DII and COPD all-cause mortality, adjusting for potential confounders identified in Model 2. To strengthen the credibility of our results, we calculated the *E*-value to evaluate the impact of unmeasured and unknown confounders on the association (31, 32).

Kaplan-Meier survival curves were constructed to visualize the cumulative survival probabilities over time for different DII tertiles. The log-rank test was used to compare survival differences among groups.

Furthermore, we conducted stratification by sex, age (20–60 years vs. >60 years), education level (≤12 years vs. >12 years), smoking status (non-smokers vs. smokers), BMI (<25 vs. ≥25 kg/m^2), CVD, hypertension, and diabetes to ascertain whether the relationship between DII and all-cause mortality in COPD is consistent across the population. Subsequently, Cox regression models were employed to assess heterogeneity and interactions among these subgroups.

Data analysis was conducted using R software (version 4.2.1; R Foundation for Statistical Computing; https://www.r-project.org/), the R survey package (version 4.1–1), and Free Statistics software (version 1.7.1; Beijing Free Clinical Medical Technology Co., Ltd.). Across all analyses, we regarded a two-sided *p*-value below 0.05 as indicative of statis-tical significance, without prior computation of statistical power due to reliance on available data.

## Results

### Baseline characteristics

This study included 1,820 participants to investigate the association between DII and all-cause mortality in patients with COPD. Table 1 presents the baseline characteristics of the participants, categorized into tertiles based on their DII scores, with 50.4% being male and an average age of 60.5 ± 12.8 years. During an average follow-up period of 7.6 years until December 31, 2019, a total of 706 participants (31.5%) died from all causes. Significant disparities were observed across the tertiles of DII in variables such as age, sex, marital status, educational level, smoking status, PIR, and levels of physical activity time (*p* < 0.05). Compared to individuals in the lowest DII tertile, those in the highest tertile were more likely to be older, female, current smokers, with tendencies towards lower educational levels, lower income, and reduced participation in physical activities Table 1.

**Table 1:** Baseline characteristics of participants according to DII score tertile.

Variables		Total		DII		*P-*value
		*T1*(-5.28-1.23)	*T2*(1.23-2.94)	*T3*(2.94-5.48)		
N	1820	607	606	607	
Age, years	60.5 (12.8)	61.3 (12.4)	60.9 (12.6)	59.2 (13.4)	0.047
Sex, *n* (%)					<0.001
Male	1025 (50.4)	403 (61.9)	338 (50.4)	284 (37.0)	
Female	795 (49.6)	204 (38.1)	268 (49.6)	323 (63.0)	
Race, *n* (%)					0.200
Non-Hispanic White	1218 (83.4)	420 (84.0)	395 (84.0)	403 (82.0)	
Non-Hispanic Black	304 (7.00)	84 (4.9)	106 (7.6)	114 (8.7)	
Mexican American	94 (1.5)	33 (1.6)	34 (1.5)	27 (1.4)	
Other Hispanic	98 (2.1)	30 (2.0)	31 (1.8)	37 (2.6)	
Other Race	106 (6.0)	40 (7.5)	40 (5.1)	26 (5.3)	
Marry, *n* (%)					0.002
Married	916 (56.8)	339 (64.9)	303 (54.6)	274 (49.5)	
Never married	129 (5.9)	43 (5.7)	40 (5.8)	46 (6.2)	
Living with partner	96 (5.8)	30 (5.0)	34 (5.8)	32 (6.8)	
Other	679 (31.5)	195 (24.4)	229 (33.8)	255 (37.5)	
Education level, *n* (%)					< 0.001
Less than 9th grade	237 (8.0)	59 (5.6)	81 (8.5)	97 (10.3)	
9-11th Grade	360 (17.1)	90 (11.4)	115 (16.0)	155 (24.8)	
High School Grad/GED or Equivalent	456 (25.8)	148 (24.5)	150 (27.9)	158 (25.2)	
Some College or AA degree	493 (28.8)	177 (29.0)	170 (28.3)	146 (29.0)	
College graduate or above	274 (20.3)	133 (29.4)	90 (19.2)	51 (10.7)	
Smoking status, *n* (%)					< 0.001
Never	285 (16.9)	99 (18.0)	94 (17.7)	92 (14.9)	
Former	880 (46.5)	329 (54.5)	296 (46.2)	255 (37.8)	
Now	655 (36.5)	179 (27.6)	216 (36.2)	260 (47.3)	
BMI (kg/m^2^)	29.3(7.4)	28.8 (6.9)	29.9 (7.9)	29.2 (7.4)	0.116
CVD					0.159
No	1231 (71.1)	428 (74.7)	412 (69.5)	391 (68.7)	
Yes	589 (28.9)	179 (25.3)	194 (30.5)	216 (31.3)	
Hypertension, *n* (%)					0.207
No	647 (40.3)	219 (42.0)	198 (36.4)	230 (42.2)	
Yes	1173 (59.7)	388 (58.0)	408 (63.6)	377 (57.8)	
DM, *n* (%)					0.391
No	1297 (76.3)	448 (78.7)	426 (74.6)	423 (75.3)	
Yes	523 (23.7)	159 (21.3)	180 (25.4)	184 (24.7)	
PIR	2.6 (1.2, 4.5)	3.3 (1.7, 5.0)	2.4 (1.2, 4.0)	1.8 (1.1, 3.6)	< 0.001
Physical activity time All-cause Mortality Alive Death FEV1/FVC PDE-4 inhibitors No Yes Mast cell stabilizers No Yes Leukotriene modifiers No Yes Inhaled corticosteroids No Yes	105.6(0.0,510.0) 1114 (68.5) 706 (31.5) 0.6 (0.1) 1813 (99.3) 7 (0.7) 1818 (99.9) 2 (0.1) 1545 (82.9) 275 (17.1) 1184 (65.3) 636 (34.7)	210.0(0.0,650.0) 398 (75.9) 209 (24.1) 0.6 (0.1) 604 (98.7) 3 (1.3) 606 (99.9) 1 (0.1) 517 (82.0) 90 (18.0) 394 (65.5) 213 (34.5)	44.9(0.0,378.0) 342 (62.1) 264 (37.9) 0.6 (0.1) 603 (99.2) 3 (0.8) 605 (99.9) 1 (0.1) 502 (81.5) 104 (18.5) 385 (64.5) 221 (35.5)	90.0(0.0,478.6) 374 (66.6) 233 (33.4) 0.6 (0.1) 606 (99.9) 1 (0.1) 607 (100) 0 (0.0) 526 (85.2) 81 (14.8) 405 (66.0) 202 (44.0)	< 0.001 0.631 0.353 0.902

Data are presented as unweighted number (weighted percentage) for categorical variables and weighted mean (standard error) for normally distributed continuous variables, or weighted median [IQR] for skewed continuous variables.

COPD, chronic obstructive pulmonary disease; T, tertiles; BMI, body mass index; CVD, cardiovascular disease; DM, Diabetes Mellitus; PIR, income to poverty ratio; DII, dietary inflammatory index.

We conducted a univariate Cox proportional hazards regression analysis to identify potential factors associated with all-cause mortality in COPD patients. The analysis revealed that age, sex, race, marital status, PIR, education level, smoking status, physical activity time, BMI, cardiovascular disease, hypertension, diabetes, and DII were significantly associated with COPD-related all-cause mortality (Supplementary Table S1). Notably, the hazard ratio (HR) for the association between DII and all-cause mortality was 1.12, indicating a statistically significant correlation (*p* = 0.001).

### Associations between DII and all-cause mortality in COPD

**Figure 2:**
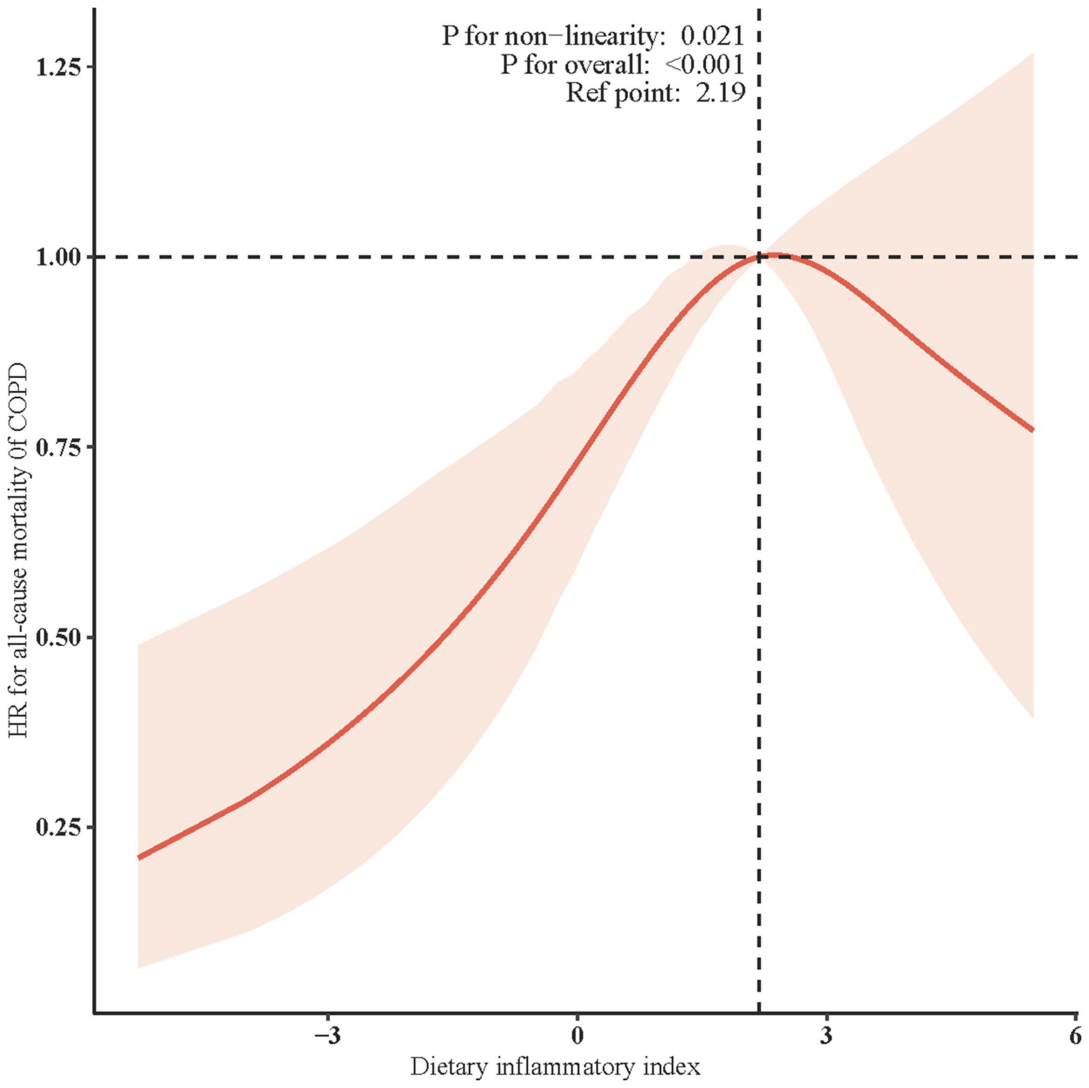
Restricted cubic spline (RCS) for the association between DII and the risks of all-cause death in patients with COPD. Solid rad line represents the smooth curve fit between variables. Blue bands represent the 95% confidence interval from the fit. All the covariates were adjusted.

Table 2 illustrates the association between DII and all-cause mortality. Both the unadjusted and multivariate-adjusted Cox regression models revealed a positive correlation, indicating that an increase in DII is associated with a higher risk of all-cause mortality. In the final model (Model 2), which adjusted for all covariates, each one-unit increase in DII as a continuous variable was associated with a 10% increase in the risk of all-cause mortality in COPD patients (HR: 1.10, 95% CI: 1.03-1.16; *p* = 0.002). When DII was stratified into tertiles, the results showed that, compared to the lowest tertile (T1), the middle tertile (T2) had a 67% higher risk of all-cause mortality (HR: 1.67, 95% CI: 1.26-2.21; *p* <0.001), while the highest tertile (T3) did not show a statistically significant increase in risk (HR: 1.30, 95% CI: 0.98-1.72; *p* = 0.074). Additionally, the trend test across tertiles showed a *p*-value of 0.082, suggesting the possibility of a nonlinear relationship (Table 2). Additionally, the restricted cubic spline model indicated a nonlinear association between DII and all-cause mortality (*p* = 0.021), with the overall association being significant (*p* < 0.001) (Figure 2). Threshold effect analysis further revealed that below a DII of 2.19, there was a significant increase in all-cause mortality risk (HR=1.19, 95% CI: 1.07-1.33; *p* = 0.002), while at or above this threshold, the increase in risk was not statistically significant (HR=0.89, 95% CI: 0.68-1.15; *p* = 0.380) (Table 3).

**Table 2:** Multivariate analysis of the association between DII and all-cause mortality of COPD.

Outcome	Events (incidence)	Non-adjusted model		Model 1		Model 2	
		HR (95 % CI)	*P*-value	HR (95 % CI)	*P*-value	HR (95 % CI)	*P*-value
DII (Continuous)	706 (38.8)	1.12 (1.07-1.18)	<0.001	1.10 (1.04-1.17)	<0.001	1.10 (1.03-1.16)	0.002
DII by tertiles							
T1 (-5.28-1.23)	209 (34.4)	1 (Reference)		1 (Reference)		1 (Reference)	
T2 (1.23-2.94)	264 (43.6)	1.90 (1.38~2.62)	<0.001	1.72 (1.28~2.32)	0.002	1.67 (1.26~2.21)	<0.001
T3 (2.94-5.48)	233 (38.4)	1.50 (1.15~1.96)	0.003	1.34 (1.01~1.77)	0.041	1.30 (0.98~1.72)	0.074
*P for trend*	706 (38.8)		0.002		0.039		0.082

Non-adjusted: no covariates were adjusted; model 1: Adjusted for age, sex, race, marry, PIR, education level; model 2: Adjusted for age, sex, race, marry, PIR, education level, smoking status, BMI, CVD, Hypertension, DM, Physical activity time. T, tertiles; OR, odd ratio; CI, confidence interval; Ref: reference; DII, dietary inflammatory index.

**Table 3:** Threshold effect analysis of the relationship of DII with all-Cause Mortality.

DII scores	Adjusted model	
	HR (95% CI)	*p*-value
<2.19	1.19 (1.07-1.33)	0.002
≥2.19	0.89 (0.68-1.15)	0.380
Log-likelihood ratio test		0.033

Adjusted for age, sex, race, marry, PIR, education level, smoking status, BMI, CVD, Hypertension, Physical activity time.

### Kaplan-Meier curves

**Figure 3:**
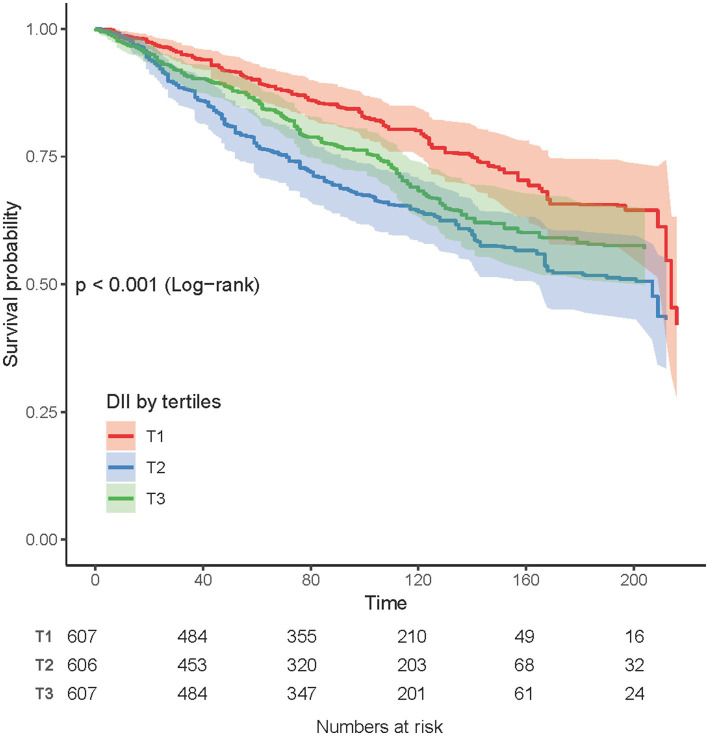
Kaplan-Meier survival curves for all-cause mortality according to Dietary DII tertiles in COPD patients.

The Kaplan-Meier curves demonstrated the association between DII and all-cause mortality in COPD patients. The lowest DII tertile group (T1) exhibited the highest survival probability throughout the follow-up period, whereas the highest DII tertile group (T3) showed a comparatively lower survival probability. The log-rank test revealed a significant difference in survival curves among the three groups (*p* <0.001; Figure 3).

### Subgroup analysis

**Figure 4:**
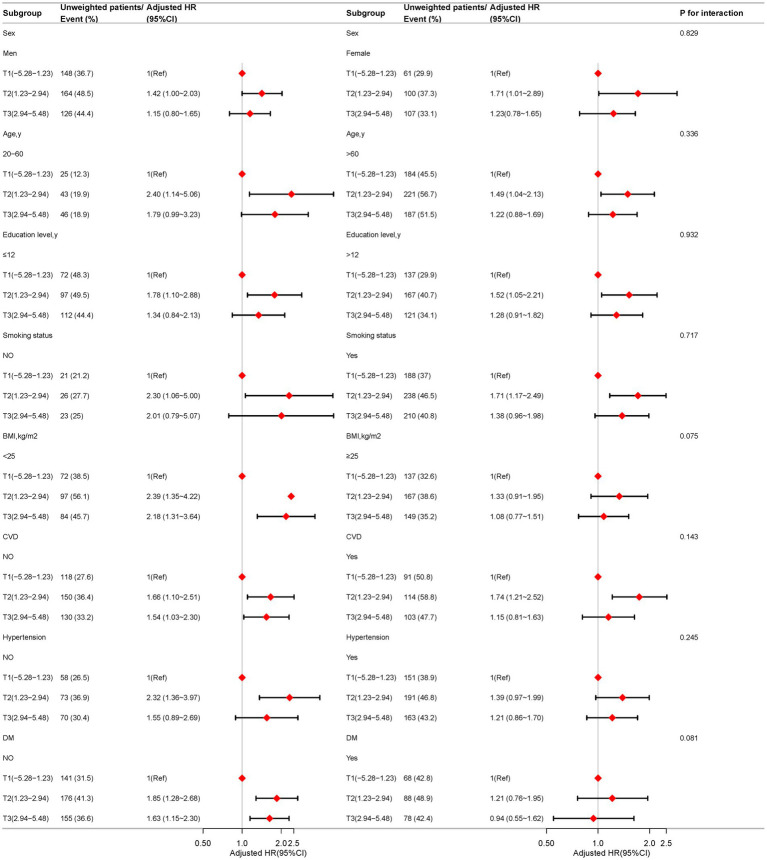
Association between the dietary inflammatory index and all-cause mortality in COPD patients based on general characteristics. Except for the stratification factor itself, the stratifications were adjusted for all variables (age, sex, race, marry, PIR, education level, smoking status, BMI, CVD, hypertension, DM, Physical activity time).

Figure 4 presents the results of subgroup analyses conducted across various demographic and health-related factors, including sex, age, educational attainment, smoking status, BMI, CVD, hypertension, and DM. These subgroup evaluations indicate that the association between DII and all-cause mortality in COPD patients remains consistent across different subgroups, with no significant interactions observed (all *p*-values > 0.05). This suggests that the relationship between DII and all-cause mortality is not significantly influenced by the variables studied. Notably, in the following specific subgroups, each unit increase in DII was significantly associated with an elevated risk of all-cause mortality: males (HR, 1.42; 95% CI: 1.00-2.03), individuals aged 20-60 years (HR, 2.40; 95% CI: 1.14-5.06), those with less than 12 years of education (HR, 2.39; 95% CI: 1.35-4.22), non-smokers (HR, 2.30; 95% CI: 1.06-5.00), individuals with a BMI below 25 kg/m² (HR, 2.32; 95% CI: 1.36-3.97), and those without cardiovascular disease (HR, 1.66; 95% CI: 1.10-2.51). In contrast, no statistically significant association was observed between DII and all-cause mortality among females, individuals over 60 years of age, those with more than 12 years of education, smokers, those with a BMI of 25 kg/m² or higher, individuals with cardiovascular disease, those with hypertension, or those with diabetes (Figure 4).

### Sensitivity analysis

To assess the robustness of the association between DII and all-cause mortality in COPD patients, we conducted an *E*-value analysis. When DII was treated as a continuous variable, the *E*-value was 1.43, indicating that any potential unmeasured confounders would need to have at least a 1.43-fold association with both DII and all-cause mortality to fully explain the observed association. Further sensitivity analysis revealed that, among different DII tertiles, the HR for the T2 group was 1.67, corresponding to an *E*-value of 2.73, while the HR for the T3 group was 1.30, with an *E*-value of 1.92. These findings suggest that the observed associations are relatively robust to potential unmeasured confounding factors across different levels of DII (Supplementary Figure 1 and Table 2).

## Discussion

To our knowledge, this is the inaugural prospective study examining the association between DII and all-cause mortality within a substantial cohort of patients with COPD. Our findings reveal a positive association between higher DII levels and increased mortality risk, consistent across various subgroups, which highlights the robustness of these outcomes. Additionally, a nonlinear relationship and threshold effect between DII levels and mortality rates offer new insights into their complex inter-action. Sensitivity analyses have further substantiated these associations, diminishing the likelihood of influence from unmeasured confounding factors. Collectively, these results suggest that the DII may serve as a crucial prognostic marker for COPD, providing essential evidence for the formulation of dietary guidelines to enhance COPD prognosis.

Our findings are consistent with several previous studies supporting the association between dietary inflammation and poor prognosis in COPD patients (13, 14, 33, 34). We observed that higher DII scores were associated with increased all-cause mortality in COPD patients, and a meta-analysis by Shivappa et al. showed that higher DII scores were similarly associated with increased all-cause mortality in the general population (13). Although we found a statistically significant difference (HR = 1.10, *p* = 0.002), the small risk difference suggests that its clinical significance may be limited. Therefore, these results should be interpreted with caution. Future large-scale studies could help further validate these findings and explore the underlying biological mechanisms. Another prospective study also found that an elevated DII score was associated with an increased risk of all-cause mortality in patients with cardiovascular disease (33). In addition, a study in patients with type 2 diabetes mellitus showed that high DII scores were associated with elevated all-cause mortality and CVD mortality (14). These findings suggest that dietary inflammation may be a common factor influencing the prognosis of various chronic diseases. Although some studies have reported inconsistent results in the association between DII and mortality, these differences may stem from differences in study design and population characteristics (35). For example, while there are studies involving a broader population (35), our study focused specifically on patients with COPD. In addition, the larger sample size and longer follow-up period of our study helped to identify significant associations that may not have been apparent in smaller or shorter-term studies. Notably, previous studies have delved into the relationship between dietary inflammation and the risk and severity of COPD (8, 36). For example, Scoditti et al. found that higher DII scores were associated with an increased risk of developing COPD (8). Another study emphasized that an elevated DII score was associated with an increased risk of lung function deterioration and acute exacerbation in COPD patients (36). These findings echo our study and further elucidate how dietary inflammation may affect the long-term prognosis of COPD patients. In conclusion, our study confirms the results of most of the existing literature and emphasizes the critical role of addressing dietary inflammation in management strategies for patients with COPD. Future studies should further explore the potential benefits of dietary interventions in improving the prognosis of COPD patients and elucidate the mechanisms involved.

Our study revealed that elevated DII scores were associated with increased all-cause mortality in patients with COPD. The DII assesses the overall dietary inflammatory load by measuring the inflammatory potential of 45 dietary nutrients and components (15). The chronic inflammatory characteristics of COPD include inflammatory cell infiltration and elevated levels of inflammatory mediators, leading to airway remodeling and lung parenchymal destruction (5). Higher DII scores suggest that the diet tends to be pro-inflammatory. The onset and progression of COPD are intrinsically linked to chronic inflammation (5). A pro-inflammatory diet may adversely affect the prognosis of COPD through multiple pathways: direct activation of inflammatory signaling pathways, such as NF-κB and MAPK, which exacerbate the systemic inflammatory load (37); alteration of the intestinal flora, and dysbiosis has been shown to be associated with systemic inflammation and COPD-related complications (38); and increase in oxidative stress and suppression of immune function, which may further exacerbate the condition of COPD (39). A high-DII diet may exacerbate COPD-related inflammation by activating pro-inflammatory signaling pathways such as NF-κB, increasing oxidative stress, and inducing gut microbiota dysbiosis (37). In contrast, a low-DII diet can exert anti-inflammatory effects by providing antioxidants, omega-3 polyunsaturated fatty acids, and maintaining gut microbiota balance (40).

In exploring the nonlinear relationship and threshold effect between DII and all-cause mortality, we found that when the DII score falls below 2.19, the mortality risk in COPD patients significantly increases. This finding suggests that varying levels of diet-induced inflammation may have different impacts on COPD prognosis. We hypothesize that a DII score close to 2.19 may represent a beneficial level of inflammation that aids in the management of COPD, while scores below or above this threshold may indicate a detrimental inflammatory state, potentially accelerating disease progression. This hypothesis aligns with the pathophysiological characteristics of COPD, where chronic inflammation leads to airway remodeling and destruction of lung parenchyma (40), whereas a moderate inflammatory response can help repair damaged tissue and defend against infections (41). However, this explanation requires further research, such as investigating changes in inflammatory markers across different DII levels.

In discussing the modifying factors that influence the prognostic impact of dietary inflammation in COPD, we note that subgroup analyses revealed that factors such as education level, smoking status, BMI, and cardiovascular and metabolic status may in-fluence the association between DII and mortality. These findings are consistent with the pathogenesis and prognostic factors of COPD. Epidemiologic studies have shown that smoking, obesity, and cardiovascular and metabolic diseases are important risk factors and prognostic indicators for COPD (10, 42). A high DII diet may indirectly affect COPD prognosis by exacerbating these factors (43). In addition, education level influences health awareness and behavior, which in turn affects disease management and prognosis (43). In conclusion, the relationship between dietary inflammation and COPD prognosis is moderated by a variety of factors, and clinicians should develop individualized dietary guidelines based on these factors.

Our study has several limitations. First, the retrospective cohort design makes it challenging to fully eliminate confounding bias and establish causality. Second, the reliance on participant recall for dietary assessment may introduce recall bias, and changes in dietary habits during follow-up were not considered. Third, the DII calculation included only 28 of the 45 originally proposed dietary parameters due to data availability, which may affect the precision of the inflammatory potential assessment. However, previous studies have validated the use of subsets of these parameters (23). Additionally, due to the limited availability of pulmonary function data, particularly post-bronchodilator measurements, and the low prevalence of certain medications with no significant differences across DII strata, these factors were not included as confounders in the analysis. Furthermore, although we found a statistically significant association between DII and all-cause mortality in COPD patients, the small risk difference, due to the limitations of sample size and follow-up duration, suggests that the clinical significance may be limited. Therefore, future prospective studies should include larger sample sizes and longer follow-up periods to enhance the generalizability and applicability of the results, and to further explore the causal relationship between dietary inflammation and COPD prognosis. Finally, like other observational studies, residual or unknown confounding effects cannot be completely ruled out. Future prospective studies with comprehensive confounder adjustment, more accurate dietary assessment, and inclusion of all 45 DII parameters are needed to further investigate the causal link between dietary inflammation and COPD prognosis.

## Conclusion

After adjusting for multiple variables, we found a significant non-linear positive correlation between DII and all-cause mortality in COPD patients. When DII was below 2.19, the risk of all-cause mortality increased significantly, while above this threshold, the risk increase was not significant. This finding suggests that excessively low DII may be associated with a higher mortality risk in COPD patients. These results emphasize the potential value of monitoring and evaluating DII in preventing mortality among COPD patients, and indicate that maintaining a moderate level of inflammation might be necessary to optimize COPD prognosis. However, this hypothesis requires further research for validation.

## Data Availability

The original contributions presented in the study are included in the article/Supplementary material, further inquiries can be directed to the corresponding author.

## Footnotes

^1^ https://www.cdc.gov/nchs/data-linkage/mortality-public.htm

^2^ https://wwwn.cdc.gov/Nchs/Nhanes/continuousnhanes/
