# Supplementary material for: Dietary inflammatory index and all-cause mortality in adults with COPD: a prospective cohort study from the NHANES 1999–2018
Source: Front Nutr. 2024 Sep 25;11:1421450. doi: 10.3389/fnut.2024.1421450 (PMC11463153; doi:10.3389/fnut.2024.1421450)
Supplement: Supplementary file 1 [file Data_Sheet_1.zip › Table S1.docx]

| Exposure | HR (95%CI) | *P-value* |  |  |  |  |  |
| --- | --- | --- | --- | --- | --- | --- | --- |
| **Age** | 1.08 (1.08,1.08) | < 0.001 |  |  |  |  |  |
| **Sex**  Male  Female | 1.0(ref)  0.9 (0.9,0.91) | < 0.001 |  |  |  |  |  |
| **Race**  Non-Hispanic White 1.0(ref) | |  |  |  |  |  |  |
| Non-Hispanic Black | 1.08 (1.08,1.08) | <0.001 |  |  |  |  |  |
| Mexican American | 0.61 (0.61,0.61) | <0.001 |  |  |  |  |  |
| Other Hispanic | 0.58 (0.58,0.59) | <0.001 |  |  |  |  |  |
| Other Race | 0.87 (0.87,0.87) | <0.001 |  |  |  |  |  |
| **Marry**  Married 1.0(ref) | |  |  |  |  |  |  |
| Never married | 0.51 (0.50,0.51) | <0.001 |  |  |  |  |  |
| Living with partner | 0.72 (0.72,0.73) | <0.001 |  |  |  |  |  |
| Other | 1.82 (1.82,1.83) | < 0.001 |  |  |  |  |  |
| **PIR** | 0.85 (0.81,0.89) | < 0.001 |  |  |  |  |  |
| **Education level**  Less than 9th grade 1.0(ref) | |  |  |  |  |  |  |
| 9-11th Grade | 0.60 (0.60,0.61) | < 0.001 |  |  |  |  |  |
| Highschooler/GED or Equivalent | 0.52(0.52,0.52) | < 0.001 |  |  |  |  |  |
| Some College or AA degree | 0.56 (0.56,0.56) | < 0.001 |  |  |  |  |  |
| College graduate or above | 0.33 (0.33,0.33) | < 0.001 |  |  |  |  |  |
| **Smoke**  Never 1.0(ref) | |  |  |  |  |  |  |
| Former | 2.59 (2.59,2.59) | < 0.001 |  |  |  |  |  |
| Now | 1.86 (1.86,1.86) | < 0.001 |  |  |  |  |  |
| **Physical activity time** | 0.9994 (0.9994,0.9994) | < 0.001 |  |  |  |  |  |
| **BMI_kg.m2** | 0.9993 (0.9929,0.993) | < 0.001 |  |  |  |  |  |
| **CVD**  No  Yes | 1.0(ref)  2.76 (2.76,2.77) | < 0.001 |  |  |  |  |  |
| **Hypertension**  No  Yes | 1.0(ref)  1.96 (1.95,1.96) | < 0.001 |  |  |  |  |  |
| **DM**  No  Yes | 1.0(ref)  1.93 (1.93,1.93) | < 0.001 |  |  |  |  |  |
| **DII** | 1.12 (1.12,1.12) | < 0.001 |  |  |  |  |  |

**Supplement Table 1**Results of Univariate Cox Regression Analysis for Factors Associated with All-Cause Mortality in COPD

**Abbreviations**: COPD, chronic obstructive pulmonary disease; T, tertiles; BMI, body mass index; CVD, cardiovascular disease; DM, Diabetes Mellitus; PIR, income to poverty ratio; DII, dietary inflammatory index
