# Supplementary material for: Dietary inflammatory index and all-cause mortality in adults with COPD: a prospective cohort study from the NHANES 1999–2018
Source: Front Nutr. 2024 Sep 25;11:1421450. doi: 10.3389/fnut.2024.1421450 (PMC11463153; doi:10.3389/fnut.2024.1421450)
Supplement: Supplementary file 1 [file Data_Sheet_1.zip › Table S2..docx]

**Table S2**. Sensitivity analysis of COPD all-cause mortality across tertiles of DII

| Outcome | ***E-value*** | | Non-adjusted Model | | Model 1 | | Model 2 | |
| --- | --- | --- | --- | --- | --- | --- | --- | --- |
|  |  |  | HR (95% CI) | *P*-value | HR (95% CI) | *P*-value | HR (95% CI) | *P*-value |
| DII(Continuous) | **1.43** |  | 1.12(1.07-1.18) | <0.001 | 1.10(1.04-1.17) | <0.001 | **1.10(1.03-1.16) 0.002** | |
| DII by tertiles | |  |  |  |  |  |  | |
| T1(-5.28-1.23) |  |  | 1(Reference) |  | 1(Reference) |  | 1(Reference) | |
| T2(1.23-2.94) | **2.73** |  | 1.90(1.38~2.62) | <0.001 | 1.72(1.28~2.32) | 0.002 | **1.67 (1.26~2.21) <0.001** | |
| T3(2.94-5.48) | **1.92** |  | 1.50(1.15~1.96) | 0.003 | 1.34(1.01~1.77) | 0.041 | **1.30(0.98~1.72) 0.074** | |
| *P for trend* |  |  |  | 0.002 |  | 0.039 | 0.082 | |

**Notes:** Non-adjusted: no covariates were adjusted; model 1: Adjusted for age, sex, race, marry, PIR, education level; model 2: Adjusted for age, sex, race, marry, PIR, education level, smoking status, BMI, CVD, Hypertension, DM, Physical activity time.

**Abbreviations:** T, tertiles; OR, odd ratio; CI, confidence interval; Ref: reference; DII, dietary inflammatory index
