# Supplementary figures and images for: Dietary inflammatory index and all-cause mortality in adults with COPD: a prospective cohort study from the NHANES 1999–2018
Source: Front Nutr. 2024 Sep 25;11:1421450. doi: 10.3389/fnut.2024.1421450 (PMC11463153; doi:10.3389/fnut.2024.1421450)

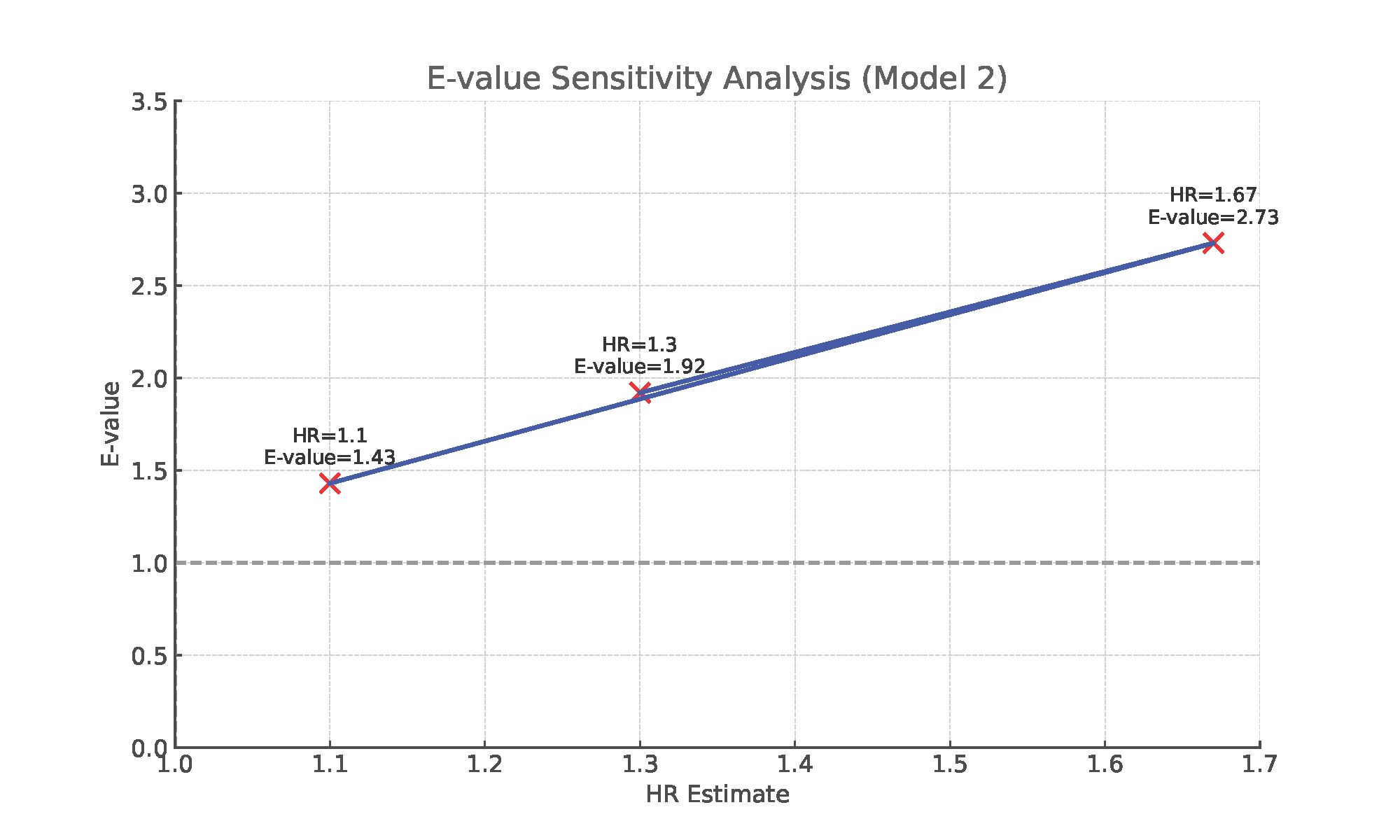

Supplement: Supplementary file 1 [file Data_Sheet_1.zip › Figure S1.tif]
